# Targeting intercellular adhesion molecule-1 (ICAM-1) to reduce rhinovirus-induced acute exacerbations in chronic respiratory diseases

**DOI:** 10.1007/s10787-022-00968-2

**Published:** 2022-03-22

**Authors:** Shakti D. Shukla, Madhur D. Shastri, Swaroop K. Vanka, Niraj Kumar Jha, Harish Dureja, Gaurav Gupta, Dinesh Kumar Chellappan, Brian G. Oliver, Kamal Dua, E. Haydn Walters

**Affiliations:** 1 Discipline of Pharmacy, Graduate School of Health, University of Technology Sydney, Ultimo, NSW 2007 Australia; 2 Faculty of Health, Australian Research Centre in Complementary and Integrative Medicine, University of Technology Sydney, Ultimo, NSW 2007 Australia; 3 School of Pharmacy and Pharmacology, University of Tasmania, Hobart, Australia; 4 Priority Research Centre for Healthy Lungs and School of Biomedical Sciences and Pharmacy, Faculty of Health and Medicine, University of Newcastle, Callaghan, NSW Australia; 5 Department of Biotechnology, School of Engineering and Technology (SET), Sharda University, Greater Noida, UP 201310 India; 6 Department of Pharmaceutical Sciences, Maharshi Dayanand University, Rohtak, Haryana 124001 India; 7 School of Pharmaceutical Sciences, Jaipur National University, Jagatpura, Jaipur 302017 India; 8 Department of Life Sciences, School of Pharmacy, International Medical University, Bukit Jalil, 57000 Kuala Lumpur, Malaysia; 9 School of Life Sciences, University of Technology Sydney, Ultimo, NSW 2007 Australia; 10 Woolcock Institute of Medical Research, University of Sydney, Sydney, NSW Australia; 11 School of Medicine, University of Tasmania, Hobart, TAS 7000 Australia

**Keywords:** Airway epithelium, Asthma, COPD, Acute exacerbations, ICAM-1, Respiratory infections

## Abstract

The chronic respiratory non-communicable diseases, asthma and chronic obstructive pulmonary disease (COPD) are among the leading causes of global mortality and morbidity. Individuals suffering from these diseases are particularly susceptible to respiratory infections caused by bacterial and/or viral pathogens, which frequently result in exacerbation of symptoms, lung function decline, frequent hospital emergency visits and increased socioeconomic burden. Human rhinoviruses (HRV) remain the major viral pathogen group implicated in exacerbations of both asthma and COPD. The rhinoviral entry into the host lung epithelium is facilitated primarily by the adhesion site (“receptor”) intercellular adhesion molecule-1 (ICAM-1), coincidentally expressed on the respiratory epithelium in these conditions. Multiple observations of increased airway ICAM-1 protein in asthmatics, smokers and smoking-related COPD have been recorded in the literature. However, the lack of robust therapies for COPD in particular has triggered a renewed interest in assessing receptor antagonism-based anti-viral strategies for treatment of intercurrent viral infections in those with pre-existing chronic lung diseases. Given the crucial role ICAM-1 plays in facilitating HRV adhesion and, thus, transmissibility to the host respiratory system, as well as the up-regulation of ICAM-1 by smoking, we summarize the role of HRV in smoking-induced COPD and especially highlight the role of ICAM-1 in epithelial viral adhesion and chronic lung disease progression. Further, the review also sheds light specifically on evolving precision therapeutic strategies in blocking ICAM-1 for preventing viral adhesion and exacerbations of COPD.

## Introduction

The overall prevalence and clinico-social burden of non-communicable chronic lung diseases (CLDs), primarily asthma and chronic obstructive pulmonary disease (COPD), is increasing constantly from an already high base. Notably, these CLDs are amongst the top five causes of morbidity and mortality worldwide, affecting more than half a billion individuals and accounting for over 3 million deaths annually (WHO 2014). Asthma is a complex and heterogeneous lung disease, predominantly characterized by airway inflammation, smooth muscle hyperplasia and airway hyperreactivity (Papi et al. 2018). Allergic asthma is the most typical asthma type that exacerbates in response to non-specific environmental triggers such as microbial infections, non-infectious exposures (mould, dust mite, pollen etc.) and host genetic makeup (Papi et al. 2018). Similarly, COPD is also a complex and heterogenous lung disease caused by long-term exposure to various toxic substances, especially cigarette smoke and biomass smoke. This repeated and chronic exposure of the airways and lungs to the deleterious constituents of noxious particles and gases results in mainly small airway remodeling and destruction, leading to the development of poorly reversible airflow obstruction (Global Strategy for the Diagnosis 2015).

Although both asthma and COPD are non-communicable diseases, there is growing evidence of an association between both asthma and COPD clinical course and several major communicable respiratory illnesses, especially COPD patients' vulnerability towards frequent viral/bacterial infections. Patients who have asthma and COPD often exhibit acute exacerbations (AE), which are described as a sudden worsening of symptoms frequently requiring specialist consultations, prescription changes, emergency GP or hospital visits and hospitalization. These AEs are significantly associated with several complications such as pneumonia, increased risk of morbidity/mortality, the triggering of a rapid decline in the lung function parameters, all of which elevate healthcare costs and compromise lifestyle (Qureshi et al. 2014). AEs occur in more than half of CLD patients despite adherence to prescribed disease management strategies.

The majority of AEs are caused by infective triggers, either bacterial or viral or both (together or sequentially). `Non-infective triggers (e.g., environmental pollutants) are also implicated, but may account for a fraction of these exacerbations. For instance, the polymerase chain reaction (PCR) based methodologies have confirmed viral pathogens in up to 77% of asthma (Johnston et al. 2005) and 60% of COPD exacerbations (Sykes et al. 2007). Importantly, Human Rhinovirus (HRV/RV) is the primary trigger for exacerbations of both asthma and COPD (Wedzicha 2004), as well as major viral pathogen implicated in childhood respiratory infections (Taylor et al. 2017). Other viruses such as coronavirus, influenza and parainfluenza viruses, and Respiratory Syncytial Virus (RSV) play a part but have a relatively minor role. Indeed, the prevalence of HRV and its viral load were higher in patients with COPD exacerbations than those with stable disease (Seemungal et al. 2001; Wedzicha 2004; George et al. 2014). Viral-induced AE episodes are also associated with more severe exacerbations and accompanying symptoms as well as greater likelihood of hospital admissions and longer recovery time (Seemungal et al. 2000). Moreover, in a clinical study involving experimental airway HRV infection in COPD patients, the HRV load correlated with sputum neutrophil counts and interleukin (IL)-8 production (Ledford et al. 2004). This indicates the activation of innate immune responses which are known to be associated with clinical presentation of HRV-induced AEs. The attempts to type HRV isolated from the airways in exacerbations of COPD or asthma have only commenced quite recently. One study reported that HRV-C was strongly associated with more severe exacerbations in asthma, including those requiring hospitalization (Cox et al. 2013). Another study identified HRV-A and HRV-C as the HRVs most predominantly isolated from exacerbating COPD or asthma patients, although HRV-A had a greater number of genotypes in both COPD and asthma (Ko et al. 2019). Moreover, there was no association observed between the various types of HRVs in either AECOPD or asthma exacerbations with hospital LOS, readmission or mortality (Ko et al. 2019). Further research is warranted to profile the cadherin-related family member 3 (CDHR3) receptor adhesion site for HRV-C in lung tissues from chronic lung diseases to devise further preventive anti-viral strategies.

A few proposed mechanisms increase viral susceptibility in patients with CRDs including immune dysregulation, mucociliary dysfunction, and notably, activated epithelial exhibiting increased expression of viral adhesion receptors. All these mechanisms combined potentially lead to more frequent episodes of “acute bronchitis” in patients with CRDs than in healthy individuals (Mallia et al. 2011). Notably, the up-regulation and modulation of surface-expressed intercellular adhesion molecule -1 (ICAM-1), either by the external stimuli (i.e., cigarette smoke) or by the HRV itself, is perhaps the predominant mechanism for HRV adherence (at least for > 60% of the HRV major group) to airway/lung epithelia. Crucially, ICAM-1 has also been shown to regulate inflammatory cell recruitment and activation, most notably macrophages and lymphocytes. Paradoxically, increased expression of ICAM-1 also seems to enhance the susceptibility to infection that is primarily viral. However, several bacterial and parasitic pathogens also utilize ICAM-1 for adhesion and invasion, such as *Haemophilus influenzae* and *Plasmodium falciparum*. Taken together, it is evident that HRV infection leads to AEs in chronic lung diseases, especially COPD as an exemplar perhaps for other CRDs, and the lack of effective preventive and/or treatment approaches for AEs currently means that more research aimed directly towards producing therapeutic blockade of this pathway is urgently warranted. Specifically, blocking viral adhesion to respiratory epithelia holds great promise to mitigate viral-induced AEs in CRDs. We will summarize the crucial role of HRV in inducing AEs in CRDs, along with the key role of ICAM-1 in facilitating the adhesion and invasion of HRV in respiratory epithelia. We will then summarize the current understanding of ICAM-1 in smokers, as well as those with established CRDs. Finally, we will briefly discuss the role of ICAM-1 in other diseases, e.g., malaria and COVID-19, and the potential of anti-ICAM-1 therapies as illustrated in mitigating microbial infections in pre-clinical models.

## Human rhinovirus (HRV)

HRV, initially isolated from individuals expressing common cold symptoms in the early 1950s, represents a serologically diverse group of pathogenic picornaviruses (> 160 serotypes) (Bochkov and Gern 2016). Depending upon the phylogenetic relationships and sequence divergence, HRVs were classified into three serotypes: HRV-A, HRV-B, and HRV-C (McIntyre et al. 2013; Kuroda et al. 2015).

HRVs are RNA viruses, containing positive-sense, non-enveloped, single-stranded RNA (ssRNA), and placed under the Picornaviridae family. Genetic makeup constitutes approximately 7200 base pairs, ranging up to 27 nm in diameter. The viral genome consists of a single functional gene, whose translated protein is cleaved by virally encoded proteases to produce 11 viral proteins (VP). Among these four proteins, VP1, VP2, VP3, and VP4 form the outer capsid that encases the genetic material. Other nonstructural proteins are involved in viral genome replication assembly. The antigenic diversity of the virus is accounted for by the proteins VP1, VP2, and VP3, while VP4 anchors the RNA core to the capsid. Each of the four capsid proteins has 60 copies, giving the virion an icosahedral structure with a canyon in VP1 that serves as a site of attachment to cell surface receptors/adhesion sites.

**Fig. 1:**
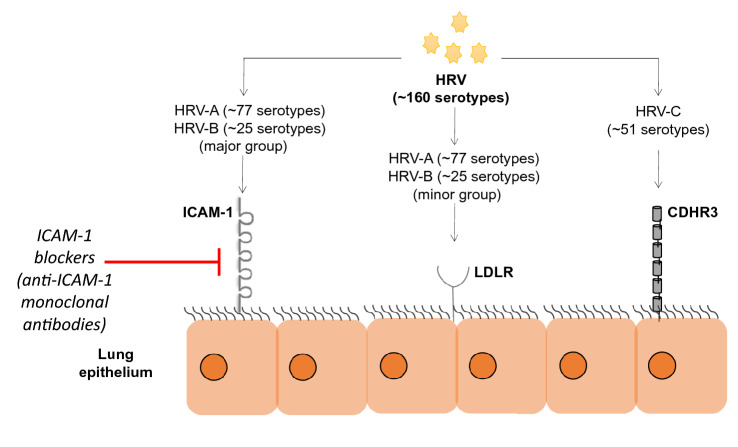
Major adhesion receptors for human rhinovirus. intercellular adhesion molecule-1 (ICAM-1) acts as the primary receptor for the major sub-group of HRV (HRV-A and HRV-B), while low-density lipoprotein receptor (LDLR) is utilized by the minor sub-group of HRV (HRV-A and HRV-B). HRV-C serotypes primarily bind to the CDHR3 receptors

As previously stated, the “major group” of known HRV serotypes, i.e. > 60% of known HRV strains, utilize the cell surface receptor ICAM-1, while other serotypes use low-density lipoprotein receptor (LDLR) as a point of entry. Studies also show that some HRVs belonging to major groups use heparan sulfate as an additional receptor (Palmenberg et al. 2010). HRV-C, in particular, uses cadherin-related family member 3 (CDHR3) to achieve cellular entry (Staunton et al. 1989; Bochkov et al. 2015) (Fig. 1).

## Respiratory infections by HRV

HRVs, especially HRV-A and HRV-C, chiefly cause upper respiratory tract (URT) infections but may also infect the lower respiratory tract (LRT), in both children and adults (Hayden 2004; Winther 2011). Several studies using molecular techniques (RT-PCR) to detect HRVs have established respiratory viral infections as a major risk factor for acute exacerbations of asthma and COPD in adults and children, frequently leading to hospital admission. HRV is also associated with wheezing illnesses in children, cardiopulmonary disease, and fatal pneumonia in immune-compromised patients. While other respiratory viruses, such as influenza virus and respiratory syncytial virus (RSV), destroy airway epithelial cells, HRV is not associated with the destruction of the epithelial lining in nasal biopsies from subjects with natural ‘colds’ (*n* = 29), determined by light or scanning electron microscopy (Winther et al. 1984).

Malmstrom et al. observed that HRV was frequently found in the lower airways in infants (*n* = 201; age 3–26 months) with recurrent respiratory symptoms. The presence of HRV was associated with increased resistance to airflow, which predicted subsequent asthma in later life (Malmstrom et al. 2006). HRV was detected by both immunohistochemistry and the indirect in-situ RT-PCR (*n* = 20) in more bronchial biopsies of asthmatics (73%) than in non-asthmatic subjects (22%). Again, the presence of HRV was associated with more airway obstruction, higher numbers of blood eosinophils and leukocytes, and eosinophilia in bronchial mucosa (Wos et al. 2008). Experimental HRV infection of human participants (*n* = 19) reported the presence of virus in cells from nasal lavage (all subjects), sputum (all subjects), bronchoalveolar lavage (26%), bronchial brushings (28%), and biopsy specimens (36%) (Mosser et al. 2005).

RNA-sequencing (RNA-seq) analysis of HRV-infected air–liquid interface differentiated human airway epithelial cell cultures from 6 asthmatic and six healthy donors identified specific sets of host gene transcriptions associated with increased inflammatory pathways, and changes in epithelial structure (remodeling) and cilium assembly and function (involving CCL5, CXCL10, CX3CL1, MUC5AC, CDHR3, and CCRL1) (Bai et al. 2015).

As stated, HRVs (HRV-A and HRV-C) are also important in triggering acute exacerbations in COPD (AECOPD). Two prospective studies of patients hospitalized with AECOPD found that picornaviruses were the most common viral infection detected by RT-PCR in nasal lavage fluid, nasopharyngeal swab, or induced sputum specimens, occurring in 36–50% of cases (Rohde et al. 2003; Kherad et al. 2010). HRV prevalence and load at exacerbation were significantly higher than in the stable state (53.3% versus 17.2%). COPD patients detected with HRV at exacerbation also had a higher exacerbation frequency per year than patients without HRV. However, secondary bacterial infection was common at about 14 days post-HRV infection (George et al. 2014). Johnston et al. assessed the importance of RV in AECOPD and reported that “cold-like” symptoms could reliably predict exacerbations in more than 80% of patients, despite less than half of patients apparently being infected. Nevertheless, patients with viral detection did present with more severe symptoms than other groups (Johnston et al. 2017).

Mallia et al. experimentally infected 13 COPD patients and 13 non-obstructed smoker controls with HRV. They reported significant reductions in post-bronchodilator peak expiratory flow and carbon monoxide diffusion capacity from baseline to HRV infection in COPD patients but no change in controls. The authors also found increased neutrophil elastase and IL-8 levels in sputum supernatants and IL-6 levels in bronchoalveolar lavage (BAL) in COPD patients infected with HRV. Although alveolar macrophages from BAL fluid have demonstrated inadequate IFN-β responses in COPD patients compared to controls, there was only a non-significant trend toward reduced IFN-α and IFN-γ production levels. Interestingly, there was no change in TNF-α levels in either group (Mallia et al. 2011). HRV also impairs the phagocytic ability of macrophage in COPD (Finney et al. 2017). Crucially, alveolar macrophages obtained from bronchoalveolar lavage fluid of COPD patients and pre-treated with HRV (P56) showed significantly diminished ability to phagocytose fluorescently labeled heat-killed *Haemophilus influenzae *but not* Streptococcus pneumoniae*. However, this was not observed in case of alveolar macrophages obtained from control participants (Finney et al. 2017).

Importantly, it is crucial to investigate and validate important biomarkers indicative of elevated risk of HRV infections in COPD, which could inform clinicians about ‘at risk’ individuals who may need personalized management strategies. For instance, Quint et al. examined serum interferon gamma-induced protein (IP)-10 as a potential biomarker of HRV infection and reported that COPD patients (*n* = 136) had higher serum IP-10 levels than age-matched controls without COPD (*n* = 70), which also correlated with sputum HRV viral load. In the same study, post 2 years of follow-up, the authors reported that serum IP-10 levels increased significantly from baseline in HRV-positive AECOPD. However, no change was observed during HRV-negative exacerbations (Quint et al. 2010). Collectively, the evidence strongly indicate the substantial diversity of HRVs and their ability to utilize host receptors such as ICAM-1 for invasion and infection.

### Intercellular adhesion molecule-1 (ICAM-1)

Human ICAM-1 (CD54; a transmembrane glycoprotein) is a member of the immunoglobulin (Ig) superfamily that contains five Ig-like domains, a transmembrane domain, and a short cytoplasmic tail (Springer 1990). It is thought to be expressed constitutively on a wide variety of cells (including leukocytes, endothelial cells and respiratory epithelial cells) (Roebuck and Finnegan 1999), but it is also further inducible by the inflammatory mediators TNF-α, IL-1β and IFN-γ, and inhibited by glucocorticoids (Hubbard and Rothlein 2000). Membrane-bound integrin receptors such as LFA-1 and Mac-1 on leukocytes, CD43, soluble fibrinogen and hyaluronan, matrix factor hyaluronan, some HRVs (~ 60% HRV-A), and *Plasmodium falciparum* malaria-infected erythrocytes serve as ligands for ICAM-1.

Physiologically, ICAM-1 plays several key roles. ICAM-1 plays a pivotal role in maintaining cell–cell interactions and facilitates leukocyte per-endothelial transmigration from blood into inflamed tissues, possibly leading to initiation of antigen-specific immune responses (van de Stolpe and van der Saag 1996; Lehmann et al. 2003). ICAM-1 functions in the T-cell-mediated host defense system, and also as a co-stimulatory molecule on antigen-presenting cells to activate MHC class II restricted T cells, and on other cell types in association with MHC class I to activate cytotoxic T cells.

The regulation of ICAM-1 at the transcriptional level has been reviewed (van de Stolpe and van der Saag 1996). It has been suggested that the expression of ICAM-1 is transcriptionally regulated through one of four pathways: NF-kB, gamma interferon-Janus kinase/signal transduction-activating transcription (JAK/STAT), mitogen-activated protein (MAP) kinase/activator protein 1 (AP1), and, indirectly, protein kinase C (PKC) (Roebuck and Finnegan 1999). The ICAM-1 promoter contains several enhancer elements, but notably nuclear factor-kappa B (NF-κB), which mediates the effects of 12-O-tetradecanoylphorbol-13-acetate (TPA), IL-1, lipopolysaccharide (LPS), TNF-α, and glucocorticoids. Because of this complexity, the regulation of ICAM-1 expression is cell-specific and depends upon the availability of cytokine/hormone receptors, signal transduction pathways, transcription factors, and post-transcriptional modification (Jahnke et al. 1995). Increased nasal ICAM-1 expression has been reported on the respiratory epithelium of allergic subjects (Gorska-Ciebiada et al. 2006). Results from in vitro primary cell culture models confirm that HRV infects lower airway tissues and suggested viral replication in lower airway epithelial cells (Schroth et al. 1999), and a significant role for ICAM-1 in the infective process (Terajima et al. 1997).

Human bronchial epithelial cells exposed to airway secretions from subjects with bronchiectasis showed increased ICAM-1 mRNA and protein levels in TNF-α-dependent manner (Chan et al. 2008). Moreover, ICAM-1 is subsequently shed by cells and is detected in plasma as soluble (s)ICAM-1, and is found to be increased in many pathological conditions, including malignancies (e.g., melanoma and lymphomas), many inflammatory disorders (e.g., asthma and autoimmune disorders), atherosclerosis, ischemia, certain neurological disorders, and allogeneic organ transplantation (van de Stolpe and van der Saag 1996).

In addition to all of the above, there is strong evidence that ICAM-1 serves as an essential receptor for a major group of human rhinoviruses (HRV) (Greve et al. 1989), for some coxsackieviruses (Marlin et al. 1990), and several bacterial pathogens including the major respiratory opportunistic pathogen, non-typeable *Haemophilus influenzae* via type IV pilus (Novotny and Bakaletz 2016). In summary, ICAM-1 is one of the most crucial conserved human receptors that is required for a number of key physiological processes, and several microbes have evolved to utilize ICAM-1 as well as other host epithelial receptors to adhere, invade and infect the host.

## Effect of cigarette smoke (CS) on ICAM-1 expression

CS exposure is one of the most important risk factors for COPD. Related to increased levels of specific cytokines and subsequent up-regulation of ICAM-1, there is a predisposition in smokers and COPD patients to frequent HRV infection and so AEs in COPD patents. Thus, small-airway epithelial cells from physiologically normal smokers (*n* = 22) showed significantly higher levels of IL-8 and ICAM-1 mRNA than the non-smokers (*n* = 17), suggesting that CS smoke is likely to increase epithelial ICAM-1 expression (Takizawa et al. 2000). Clinical studies have demonstrated an elevated level of serum soluble (s)ICAM-1 in COPD-smokers (*n* = 142) compared to non-COPD active smokers (*n* = 55) (Lopez-Campos et al. 2012). An in vitro study using primary human bronchial epithelial cells (HBEC) from COPD-smokers and non-smoking control participants reported a significantly increased release of sICAM-1 and IL-1β in cells exposed to CS, compared to control air-exposed cells. In addition, exposure to CS increased permeability of cell culture layers from currently smoking participants (Rusznak et al. 2000). As stated in earlier sections, IL-1β further increases ICAM-1 expression on both airway epithelial cells and endothelial cells.

HRV itself upregulates membrane-bound ICAM-1 expression via a NF-κβ-dependent mechanism in both primary bronchial epithelial cells (12-fold) and A549 cloned respiratory epithelial cells (threefold) (Papi and Johnston 1999). Anti-ICAM-1 antibody (14C11) administered topically or systemically, has been shown to inhibit major group HRV replication in vitro, as well as HRV-induced inflammation, pro-inflammatory cytokine induction and lung virus RNA levels in a mice model (Traub et al. 2013), confirming a potentially crucial role for ICAM-1 as a specific viral facilitator. However, interestingly and perhaps disappointingly, anti-ICAM-1 antibody (14C11) did not prevent cell adhesion via human ICAM-1/LFA-1 interactions in vitro, suggesting the epitope targeted by 14C11 was specific for viral entry.

Although most attention has been on HRV, evidence indicates that ICAM-1 also serves as an adhesion molecule for *Haemophilus influenzae* (NTHi, via bacterial P5 fimbriae), which is the main bacterial pathogen in COPD, again especially in AECOPD (Sethi and Murphy 2008). Thus, ICAM-1 is an attractive target to block not only for virus-receptor binding but also to check ICAM-1-mediated NTHi adhesion to respiratory cells.

## The role of ICAM-1 in asthma

The role of ICAM-1 in asthma has been extensively studied since the 1990s. Wegner and colleagues first discovered the contribution of ICAM-1 to eosinophil migration and increased airway responsiveness (Wegner et al. 1990). Several subsequent studies further validated the potential link between ICAM-1 expression and asthma pathogenesis (Ciprandi et al. 1994; Stanciu and Djukanovic 1998; Lin et al. 2003). ICAM-1 and LFA-1 protein are constitutively expressed on the membrane of various human cells, including T cells and bronchial endothelial cells (Bentley et al. 1993). The expression of ICAM-1 is known to be elevated in asthma, and this favors the binding of ICAM-1-positive cells to LFA-expressing cells (Drake et al. 2018). The adherence of ICAM-1 and LFA-1 proteins then results in uropod formation; these contain regrouped adhesion molecules that facilitate T-cell capture and transmigration into the vascular lumen, resulting in increased T-cell activation and inflammation (Drake et al. 2018). The transmigration of T cells then results in shedding of soluble(s)-ICAM-1 proteins into the blood circulation, which serves as negative feedback for the ICAM-1/LFA-1 binding process and is reported to inhibit further aggregation and transmigration of T cells into the vasculature (Drake et al. 2018). On the contrary, several studies have questioned the potential role of sICAM-1 as an antagonist for ICAM-1/LFA-1-induced inflammation in asthma, primarily because sICAM-1 is also known for its pro-inflammatory properties which play a significant role in diseases like systemic inflammatory response syndrome and gram-negative pneumonia (Mendez et al. 2011; de Pablo et al. 2013). In addition, one study has previously demonstrated that sICAM-1 facilitates the secretion of eosinophil cationic protein and worsens inflammation in asthma, which is contradictory to its proposed antagonist effect on asthma exacerbations (Chihara et al. 1995).

A transmembrane protein known as orosomucoid-like (ORMDL) 3 has gained significant interest in reinforcing ICAM-1-associated asthmatic inflammation. ORMDL3 is encoded by the *ORMDL* gene family which is strongly correlated with asthma in genome-wide association studies (Galanter et al. 2008). Importantly, ORMDL3 is expressed on lung, pancreas and kidney (Miller et al. 2012), and notably, one study reported that ORMDL3 enhanced the replication of HRV in cultured human lung epithelial cells, suggesting a potential role in viral-induced asthma AEs (Liu et al. 2020). A possible direct link between ORMDL3 and ICAM-1 may also exist. For instance, Zhang et al. recently reported that *ORMDL3* knock-down in cultured lung epithelial cells reduced the expression of ICAM-1(Zhang et al. 2019). Besides ICAM-1, the levels of other inflammatory markers, including IL-1β, were found to be downregulated after *ORMDL3* knock-down, indicating its potential role in facilitating ICAM-1-induced asthmatic inflammation (Zhang et al. 2019). Further studies are warranted to validate these findings especially *in vivo* and *ex vivo* settings. Nevertheless, the potential role of ICAM-1 in asthma is now widely accepted, so the development of therapeutic approaches targeting these proteins may well be beneficial to reduced inflammation, AHR and asthma exacerbations.

## ICAM-1 expression in COPD

Despite the likely importance of ICAM-1 in AECOPD, the expression pattern of this viral adhesin has only recently been elucidated in human COPD airways (Shukla et al. 2017a, b). Briefly, the protein expression of ICAM-1 was upregulated throughout the respiratory tract (both large and small airway epithelium) in smokers but was especially marked in subjects with COPD, even when mild (Shukla et al. 2017a, b). Notably, increased ICAM-1 expression was also found in goblet cells and submucosal glands in the large airway of smokers, but especially so in COPD patients (Shukla et al. 2017a, b), which showed greater staining intensity. Moreover, ICAM-1 expression, both at the mRNA and protein level, was upregulated in cultured bronchial epithelial cells (BEAS-2B) exposed to cigarette smoke extract (Shukla et al. 2017a, b).

These findings taken as a whole may be crucial for understanding the vulnerability of smokers and especially COPD patients to airway viral infections, specifically with major group HRVs (group A and B), but also potentially with NTHi. Another study reported the key role of ICAM-1 in the clearance of NTHi from lungs in an elastase-induced experimental model of COPD/emphysema. Mice treated with elastase demonstrated diminished epithelial ICAM-1 protein levels when compared to control mice, presumably a direct effect of the proteinase, accompanied by increased aggregation of NTHi in elastase affected areas (Pang et al. 2008).

## The role of ICAM-1 in other diseases

Remarkably, ICAM-1 also plays a vital role in various other infectious diseases, including tuberculosis and malaria. For instance, the addition of M5 protein that adheres explicitly to ICAM-1 and ICAM-4, or siRNA targeting ICAM-1 decreased infection of cultured THP-1 (a human monocytic leukemia cell line) and murine peritoneal macrophages by *Mycobacterium tuberculosis* (Bhalla et al. 2015). In the same study, the authors also showed that M5 protein blocked the parasite translocation of red blood cells by *Plasmodium falciparum,* irrespective of whether the strain is drug-susceptible or resistant (Bhalla et al. 2015). Quite recently, ICAM-1/LFA-1 complex has been shown to play an important role in transmission of Murine Leukemia virus between leucocytes, thus, exploiting the host proteins involved in intercellular interactions and barrier function (Engels et al. 2022). Collectively, it is evident that various microbes exploit host ICAM-1/LFA-1 in cell-to-cell transmission and infection, thus, providing a robust pathway to mitigate microbial infection and persistence. These mechanism, however, should be amenable to novel therapeutic interventions.

## ICAM-1 as a potential anti-adhesion therapeutic target

The crucial role of ICAM-1 in facilitating some HRV adhesion has led to investigations in blocking this interaction in the context of HRV infections in in vitro cell culture and pre-clinical animal models (Shukla et al. 2017a, b). One study reported a series of novel small compounds, especially “compound 6”, which inhibited viral multiplication (HRV-A/HRV-B). Furthermore, in silico analyses suggest that the activity of compound 6 was indeed similar to that of pleconaril (Kim et al. 2017). Another compound referred to as 3 k was highly efficacious against multiple strains of HRV in in vitro models (Kim et al. 2018).

Another notable anti-adhesion therapy is highly specific monoclonal antibody (mAB) that could block the adhesion interaction between HRV and ICAM-1. For instance, although intranasal administration of rhinovirus receptor murine monoclonal antibody (RRMA, or mAb 1A6) at a dose of 135 μg/subject in human participants did not reduce the rate of infection, a higher dose (1 mg/subject) was associated with delayed shedding of virus and the onset of “cold-like” symptoms. The higher dosage of RRMA was also associated with a reduction in viral titers (Hayden et al. 1988). In addition, mAb 1A6 was shown to prevent HRV infection both in tissue culture and in an animal model of HRV infection (Colonno 1992).

Current research is focused on effective compounds that could inhibit HRV infectivity in pre-clinical cell culture models. For instance, a novel compound, (E)-(S)-4-((S)-2-pent-4-ynoylamino)-5-((S)-2-oxo-pyrrolidin-3-yl)-pent-2-enoic acid ethyl ester, irreversibly inhibited the HRV 3C protease (Patick et al. 2005). More importantly, this compound was found to be active against a wide range of HRV serotypes (35), as well as HRV clinical isolates (5). The inhibitory activity extended to other picornaviruses (8) (mean EC50: 50–75 nM). The compound was also found safe and non-toxic post a single oral dose (~ 2000 mg) administration in healthy participants (Patick et al. 2005).

Although the investigation of novel anti-ICAM-1 therapies are challenging, it is now essential in order to better manage chronic infection in the airways of CRD patients, as well as individuals with COPD experiencing frequent AEs.

## Pharmacological properties of anti-ICAM-1 therapies

The results from pre-clinical models have indicated a strong potential for ICAM-1 blockage of HRV infections. Notably, the pharmacological parameters of these anti-ICAM-1 compounds seem to be excellent. For instance, one compound, referred in the previous section as 3 k, exhibited low systemic clearance (0.158 Lh^−1^ kg^−1^) and acceptable bioavailability in the oral cavity (27.8%) (Kim et al. 2018). In an in vivo murine model of RV infection, the intravenous dosing of anti-ICAM-1 monoclonal antibody (14C11) mitigated HRC-16 induced inflammation (total cells, neutrophils, lymphocytes and macrophages in the bronchoalveolar lavage fluid) in the lung and cytokine release (Traub et al. 2013). Remarkably, these reductions in inflammatory markers were observed up to seven days post administration of a single dose of anti-ICAM-1 monoclonal antibody (Traub et al. 2013). The clinical and anti-viral potential of such adhesion receptor blocking compounds could lead to novel therapies for managing HRV infections in humans, especially in those with underlying chronic lung diseases or a compromised immune system. This would have the added advantage of prophylactic, long-term protection against the HRV, in particular preceding vulnerable seasonal changes.

## Clinical implications of blocking ICAM-1

The clinical utility of ICAM-1 in modulating HRV-induced lung inflammation and associated symptoms has been analyzed in a handful of clinical trials. Turner and colleagues (Turner et al. 1999) evaluated the efficacy of recombinant, soluble ICAM-1 (tremacamra or BIRR 4; 367 µg of tremacamra per nostril per dose for a total of 4.4 mg/day administered as inhaled solution/powder) in a randomized, double-blind, placebo controlled clinical trial. The authors found significant reductions in clinical symptoms (symptom score, clinical colds and nasal mucus) in the individuals who were experimentally infected with rhinovirus type 39 and received tremacamra, either pre- or post-inoculation of viral pathogen (100–300 median tissue culture infective dose administered as 2 inocula of 250 µL per nostril) (Turner et al. 1999). Another approach could be the utilization of gene therapy with downregulating the expression of ICAM-1 on respiratory epithelial cells (Othumpangat et al. 2016). Although not studied clinically and not in the context of HRVs, the downregulation of ICAM-1 by ICAM-1 siRNA on human bronchial epithelial cells resulted in alteration of influenza virus copy numbers, suggesting the crucial role of ICAM-1 in this viral infection (Othumpangat et al. 2016). Both anti-ICAM-1 monoclonal antibodies and/or soluble ICAM-1 protein could offer effective anti-viral approaches to protect against HRV in vulnerable populations. Once the complete pharmacological profiling is complete, this approach could offer a new avenue for prophylactically managing HRV infections in humans, and especially in patients with underlying lung conditions.

## Summary and concluding remarks

HRV is major microbial pathogen implicated etiologically in virally-induced acute exacerbations (AEs) in chronic lung diseases, especially COPD and asthma, but probably also in other chronic respiratory diseases such as Idiopathic Pulmonary Fibrosis (IPF). This poses huge healthcare and economic burden on most countries globally. Crucially, there are as yet no effective prophylactic treatments for preventing HRV infections in these highly susceptible populations, as well as no definitive means to mitigate the primary infection or secondary bacterial infections in high risk patient cohorts. ICAM-1, which acts as a primary adhesion site for the HRV, could be effectively blocked to prevent viral-binding to host cells, thus preventing viral-induced infections and associated pathology in patients with COPD/asthma (as well as IPF etc.). There is also potential to combine anti-ICAM-1 therapies with anti-PAFr therapies, that could theoretically then prevent both viral and secondary bacterial infections in highly susceptible lung disease patients. Further research should also focus on characterizing the adhesion receptors for minor group HRVs, such as LDLRs, and other potential receptors, particularly in smokers and patients with COPD and asthma. A combination of ICAM-1 blockers and inhibitors of viral replication could be ideally suited for preventing viral exacerbations in patients with chronic respiratory disease, including smoking-related COPD.

## Data Availability

Enquiries about data availability should be directed to the authors.
