# Correction to: Targeting intercellular adhesion molecule-1 (ICAM-1) to reduce rhinovirus-induced acute exacerbations in chronic respiratory diseases

**DOI:** 10.1007/s10787-022-01018-7

**Published:** 2022-07-09

**Authors:** Shakti D. Shukla, Madhur D. Shastri, Swaroop K. Vanka, Niraj Kumar Jha, Harish Dureja, Gaurav Gupta, Dinesh Kumar Chellappan, Brian G. Oliver, Kamal Dua, E. Haydn Walters

**Affiliations:** 1 Discipline of Pharmacy, Graduate School of Health, University of Technology Sydney, Ultimo, NSW 2007 Australia; 2 Faculty of Health, Australian Research Centre in Complementary and Integrative Medicine, University of Technology Sydney, Ultimo, NSW 2007 Australia; 3 School of Pharmacy and Pharmacology, University of Tasmania, Hobart, Australia; 4 Priority Research Centre for Healthy Lungs and School of Biomedical Sciences and Pharmacy, Faculty of Health and Medicine, University of Newcastle, Callaghan, NSW Australia; 5 Department of Biotechnology, School of Engineering and Technology (SET), Sharda University, Greater Noida, UP 201310 India; 6 Department of Pharmaceutical Sciences, Maharshi Dayanand University, Rohtak, Haryana 124001 India; 7 School of Pharmaceutical Sciences, Jaipur National University, Jagatpura, Jaipur 302017 India; 8 Department of Life Sciences, School of Pharmacy, International Medical University, Bukit Jalil, 57000 Kuala Lumpur, Malaysia; 9 School of Life Sciences, University of Technology Sydney, Ultimo, NSW 2007 Australia; 10 Woolcock Institute of Medical Research, University of Sydney, Sydney, NSW Australia; 11 School of Medicine, University of Tasmania, Hobart, TAS 7000 Australia

## Correction to: Inflammopharmacology (2022) 30:725–735 10.1007/s10787-022-00968-2

The 7th author name has been incorrectly published in the original publication. The complete correct name should read as follows 

Dinesh Kumar Chellappan

The original article has been corrected.

